# Clinical Predictors of Long-term Success in Ultrasound-guided High-intensity Focused Ultrasound Ablation Treatment for Adenomyosis: A Retrospective Study

**DOI:** 10.1097/MD.0000000000002443

**Published:** 2016-01-22

**Authors:** Xin Liu, Wei Wang, Yang Wang, Yuexiang Wang, Qiuyang Li, Jie Tang

**Affiliations:** From the Department of Ultrasound, Chinese PLA General Hospital, Beijing, China.

## Abstract

The long-term outcomes of ultrasound-guided high-intensity focused ultrasound (USgHIFU) ablation treatment for adenomyosis and the relevant factors affecting the durability of symptom relief were assessed in this study.

A total of 230 women with adenomyosis who were treated with USgHIFU ablation between January 2007 and December 2013 were retrospectively analyzed. The contrast-enhanced ultrasonography (CEUS) was performed immediately after the treatment to evaluate the ablation effect, and the nonperfused volume (NPV) ratio was then calculated. Regular follow-up was conducted and the visual analog scale (VAS) score was used to assess the changes in dysmenorrhea. The effect of treatment was evaluated after an average follow-up length of 3 months and the factors affecting clinical success and symptom relapse were identified.

Of the 230 treated patients, 208 (90.4%) were followed up regularly, with a median follow-up length of 40 months (range, 18–94 months). Mean value of the NPV ratio calculated immediately after the treatment was 57.4 ± 24.4%. Varying degrees of symptomatic relief of dysmenorrhea based on the VAS scores were observed in 173 (83.2%) patients and 71.0% of the patients were asymptomatic during follow-up. Women with higher NPV ratio (OR = 0.964, 95% CI = 0.947–0.982, *P* = 0.000) and older age (OR = 0.342, 95% CI = 0.143–0.819, *P* = 0.016) were more likely to achieve clinical success. Dysmenorrhea recurred in 45 (26%) out of 173 cases; the median recurrence time was 12 months after treatment. The lower BMI (OR = 1.221, 95% CI = 1.079–1.381, *P* = 0.001) and the higher acoustic power (OR = 0.992, 95% CI = 0.986–0.998, *P* = 0.007) were associated with less risk of relapse. Twelve of the 14 patients who were retreated by USgHIFU ablation after experiencing dysmenorrhea recurrence achieved clinical success.

USgHIFU ablation is an effective uterus-conserving treatment for symptomatic adenomyosis with an acceptable long-term success rate. Higher chance of clinical success can be achieved in patients with larger NPV ratio and older age, whereas higher BMI and lower acoustic power may result in a higher chance of recurrence. These factors are helpful in selecting suitable patients for USgHIFU and in predicting the durability of symptom relief.

## INTRODUCTION

Adenomyosis is a benign uterine disease characterized by the invasion of endometrial glands and stroma in the uterine myometrium, resulting in dysmenorrhea and menorrhagia. Historically, hysterectomy is considered as the definitive treatment for adenomyosis. However, for women who wish to preserve uterus, there is an increasing array of conservative therapies such as oral hormonal medications, levonorgestrel-releasing intrauterine system, uterine-sparing surgery, uterine artery embolization (UAE), and high-intensity focused ultrasound (HIFU) ablation, which are alternatives to hysterectomy for the relief of adenomyosis symptoms.^1,2^

HIFU ablation is a noninvasive tissue-ablation technique for the treatment of solid tumors.^3^ Previous studies showed that HIFU ablation is a safe and effective treatment for adenomyosis;^4–9^ however, little studies have focused on the long-term value of the HIFU ablation treatment for >3 years. In addition, relevant factors that can help in selecting appropriate candidates for the treatment are still undefined. The aims of our study are to assess the long-term outcomes of USgHIFU ablation for adenomyosis and to define the parameters associated with the clinical success and symptom relapse.

## MATERIALS AND METHODS

### Patients

The study was approved by the Ethics Committee and Institutional Review Board of Chinese PLA General Hospital, Beijing, China. Inclusion criteria for this study were (1) magnetic resonance imaging (MRI) evidence of the typical features of adenomyosis, with depth >3 cm; (2) severe dysmenorrhea and/or menorrhagia; (3) no desire to bear children; (4) premenopausal women; (5) ability to communicate with the researchers during the treatment. Patients with any of the following criteria were excluded from the study: (1) inability to lie in a prone position for ∼2 h; (2) extensive abdominal scars in the proposed acoustic pathway; (3) acute pelvic inflammatory disease.

### Preoperative Imaging Examination

All patients underwent MRI before treatment to determine the location, dimension, and the type of adenomyosis on T2-weighted images. According to the baseline,^10–12^ the type of adenomyosis was classified as focal and diffuse. The length, width, and depth of the uterus and the adenomyotic lesions were measured on T2-weighted images. The largest distance from the adenomyotic lesions dorsal side to shin was also measured. The uterus and adenomyotic lesions volumes were calculated according to the following equation: V=0.5233 × length × width × depth.^13^ All of the measurement procedures were carried out by an experienced radiologist. In addition, pretreatment CEUS was performed to evaluate the blood supply of the targeted adenomyotic lesions. The ultrasound contrast agent used in this study was SonoVue (Bracco, Milan, Italy). A 1.2-mL bolus of SonoVue was injected intravenously through a cubital vein followed by a 5-mL saline flush to ensure that no residual contrast remained in the intravenous catheter. Imaging started immediately after injection and lasted 2 to 4 min.

### Equipment

USgHIFU ablation was performed with the JC HIFU system (model JC; Chongqing Haifu Technology, Chongqing, China). The therapeutic ultrasound energy is produced by a 20 cm diameter transducer with a focal length of 140 mm operating at a frequency of 0.8 MHz. The focal region is 9.8 mm along the beam axis and 1.3 mm in the transverse direction. In the center of therapeutic transducer, a diagnostic ultrasound probe (3.5–5.0 MHz) is used to provide real-time imaging during the treatment. The energy emission and therapeutic transducer movement were controlled by the computer system.

### USgHIFU Ablation

Patients were placed above the degassed water in the prone position on the system. To provide a proper acoustic pathway, degassed water was infused to the bladder through the catheter to control the volume of bladder. Different size degassed water balloons were used to push away the bowel from the acoustic pathway when necessary. Guided by previously acquired MRI, the real-time ultrasound system was used to identify the location and rough margin of the targeted adenomyotic lesions. The focused spot was positioned at least 1.5 cm from both serosal layer of the uterus and the endometrium. An acoustic power of 286 to 520 W was used. Any discomfort during treatment was requested to be reported. The focused spot was moved when sacral nerve irritation was observed and the treatment was terminated after the gray-scale of the targeted lesions was changed.

### Post-treatment CEUS Examination

Post-treatment CEUS was performed immediately after USgHIFU ablation to evaluate therapeutic effectiveness. The injection route of the ultrasound contrast agent was the same as pretreatment CEUS. Compared with the pretreatment CEUS, the area of no contrast agent perfusion in the arterial phase and the parenchymal phase in the treated area was necrotic region. The length, height, and width of the necrotic region was measured and the volume was calculated using the same equation for a prolate ellipsoid.^13^ The NPV ratio was calculated according to the following equation: NPV% = the volume of the necrotic tissue/the volume of the targeted lesions × 100%.

### Post-treatment Follow-Up

Patients were interviewed by phone after 3 and 6 months and yearly thereafter to assess symptom relief. The intensity of dysmenorrhea was assessed by the visual analog scale (VAS) score ranging from 0 to 10.^4,7^ The efficacy of treatment was evaluated at 3 months after treatment using the following method: (1) inefficient: with a VAS score reduction of ≤ 20%; (2) partial relief: with a VAS score reduction of 20% to 50%; (3) significant relief: with a VAS score reduction of 50% to 80%; (4) complete relief: with a VAS score reduction of ≥ 80%. A result of either (2) (3) or (4) was defined as clinical success. A clinical relapse was defined once the VAS score achieved 3 months after the treatment reached >80% of the pretreatment value. Any complications during the follow-up were also recorded and graded according to the Society of Interventional Radiology (SIR).^14^

### Statistical Analysis

Normally distributed data was reported as the mean ± SD, whereas non-normally distributed data was reported as the median and interquartile range.

Multivariate logistic regression models were established with the enter regression method to identify parameters correlating with the clinical success. Multiple liner regression models were established with a stepwise regression method to identify parameters correlating with the NPV ratio. Variables included in the models were all variables found significant in the univariate analysis. Receiver operating characteristic curve analysis was used to determine an NPV ratio cutoff point percentage to predict clinical success. The Kaplan–Meier method was used to estimate the cumulative rate of freedom from recurrence. Relationships between variables and recurrence were evaluated using Cox proportional hazards models.^15^For statistical analysis, IBM SPSS 17.0 was used. The level of statistical significance was *P* < 0.05.

## RESULTS

### Baseline Characteristics of the Patients

From January 2007 to December 2013, 230 patients underwent USgHIFU ablation treatment. During the follow-up, 22 patients become unreachable and then excluded from the statistical analysis. Median follow-up of the remaining 208 women was 40 months (range, 18–94 months). All the patients had dysmenorrhea and 37 of them also had anemia due to menorrhagia. The baseline characteristics of the patients were summarized in Table 1.

**TABLE 1:**
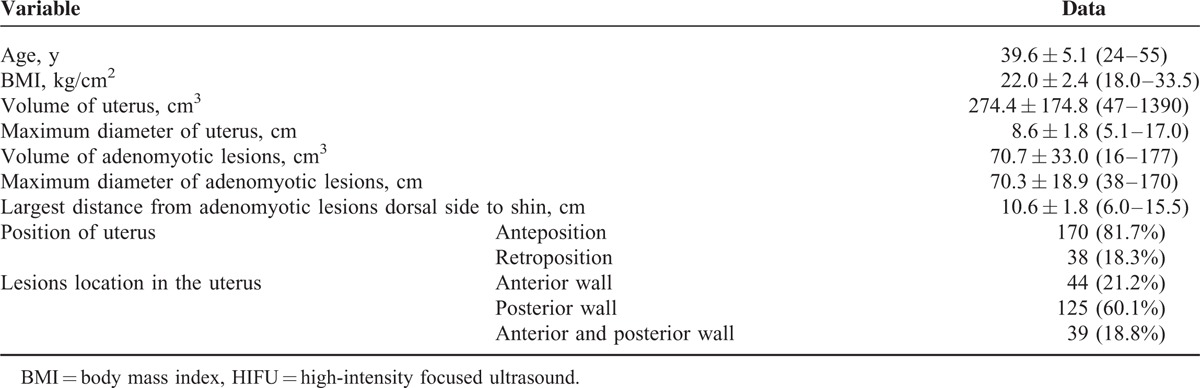
Baseline Characteristics of the Patients Before HIFU Treatment

### Treatment

**FIGURE 1:**
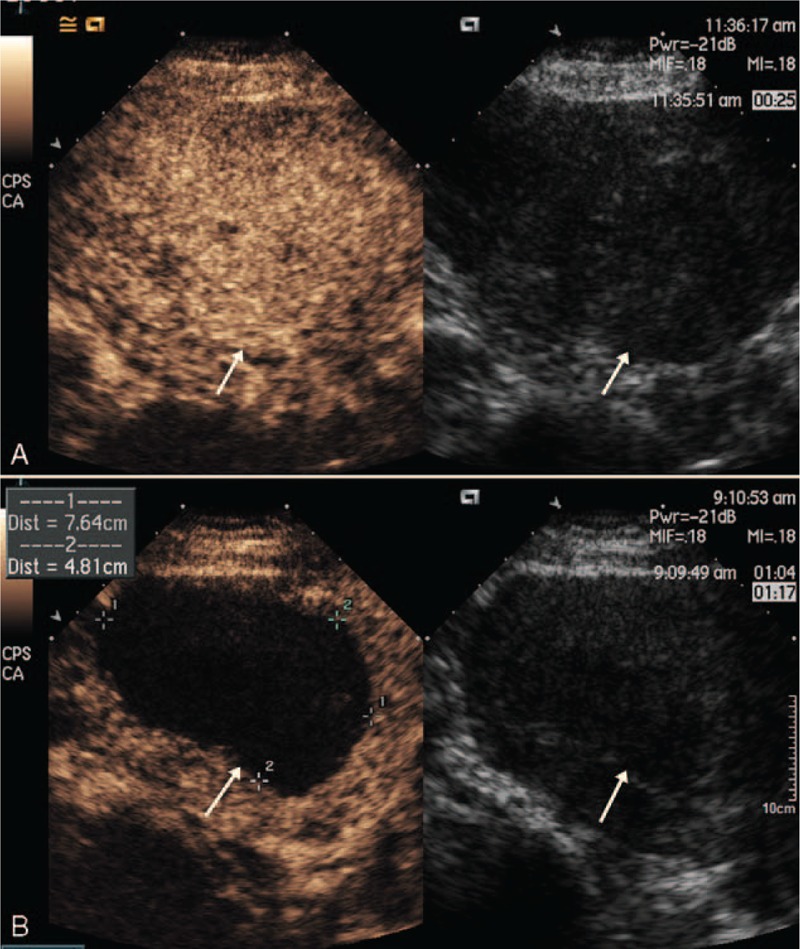
A 45-year-old woman who underwent USgHIFU for symptomatic adenomyosis. (A) CEUS obtained before USgHIFU showed inhomogeneous enhancement (arrows) of the posterior wall of the uterus. (B) Immediate CEUS obtained after treatment showed a nonenhanced region (191 cm^3^) and an NPV ratio of 68.7% in the treated adenomyosis. The margin of the nonenhanced region (arrows) was showed at the ablated region. CEUS = contrast-enhanced ultrasonography, NPV = nonperfused volume, USgHIFU = ultrasound-guided high-intensity focused ultrasound.

All the patients completed USgHIFU ablation on an outpatient basis. The mean acoustic sonication power was 485 ± 55 W (range, 286–520 W); the median treatment time was 64 min (interquartile range, 47–91 min); the median sonication time was 1135 s (interquartile range, 769–1561 s); the mean nonperfumed volume (NPV) was 72.8 ± 57.3 cm^3^ (range, 1–340 cm^3^); the mean NPV ratio was 57.39 ± 24.39% (range, 1–100%) (Figure 1).

### Symptom Relief

Among the 208 patients, 173 (83.2%) achieved clinical success with varying degrees of alleviation of their symptom of dysmenorrhea. The VAS scores showed a considerable reduction from a baseline mean of 8.9 ± 1.4 to 3.4 ± 2.9, 3 months later (*P* < 0.001). The overall effectiveness rates were as follows: complete relief in 63 (30.3%); significant relief in 97 (46.6%); partial relief in 13 (6.3%). A total of 71% patients were asymptomatic during follow-up, of whom 19 patients had been menopausal. In addition, the hemoglobin increased to the normal level in 35 patients after treatment.

### Factors Associated With Clinical Success

An association between the NPV ratio and clinical success was found in multivariate analysis (Table 2). The larger NPV ratio were associated with higher chance of success (OR = 0.964; 95% CI = 0.947–0.982; *P* = 0.000). By analyzing the NPV ratio and treatment outcome of each patient using the receiver operating characteristic curve, an NPV ratio cutoff point of 39.2% was determined to be predictive of clinical success (area under the curve = 0.722; 95% CI = 0.623–0.820; *P* = 0.000). NPV ratios of ≥39.2% resulted in an 89.9% clinical success rate as compared to the group with NPV <39.2% whose clinical success rate was 62.0%. In the univariate analysis, several parameters showed significant correlation with the NPV ratio: diffused lesions had lower NPV ratios than focal lesions (49.6% versus 59.2%, respectively). Uterus volume and treated adenomyotic lesions volume had a negative correlation with the NPV ratio (−0.138 and − 0.205, respectively). NPV and mean acoustic intensity had a positive correlation with the NPV ratio (0.357 and 0.140, respectively). All of these 5 factors were included into the liner regression models. Four factors were ultimately included into the multiple regression models: the type of adenomyosis (*P* < 0.01), treated adenomyotic lesions volume (*P* < 0.001), NPV (*P* < 0.001), and mean acoustic intensity (*P* < 0.01).

**TABLE 2:**
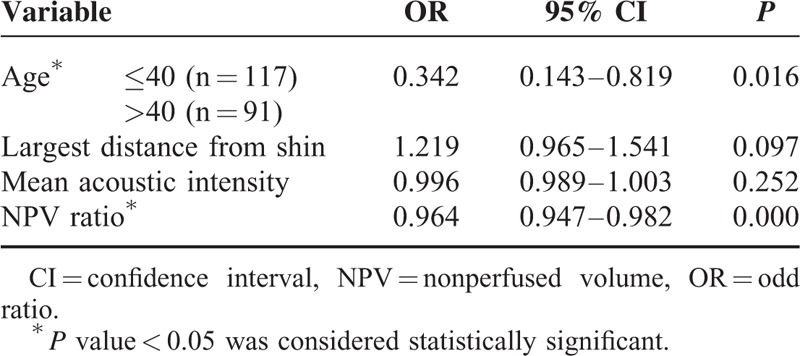
Multivariate Analysis of Parameters Associated With Treatment Outcome

The average age of the entire cohort was 40 years (median, 40 years; range, 24–55 years); age of ≥40 years resulted in a higher chance of clinical success rate (89.0%) than the group with age <40 years (78.6%) (OR = 0.342; 95% CI = 0.143–0.819; *P* = 0.016).

In both univariate and multivariate analysis, the BMI, volume of uterus, position of uterus, lesions location in the uterus, and largest distance from shin showed no significant correlation with clinical success.

### Symptom Recurrence

**FIGURE 2:**
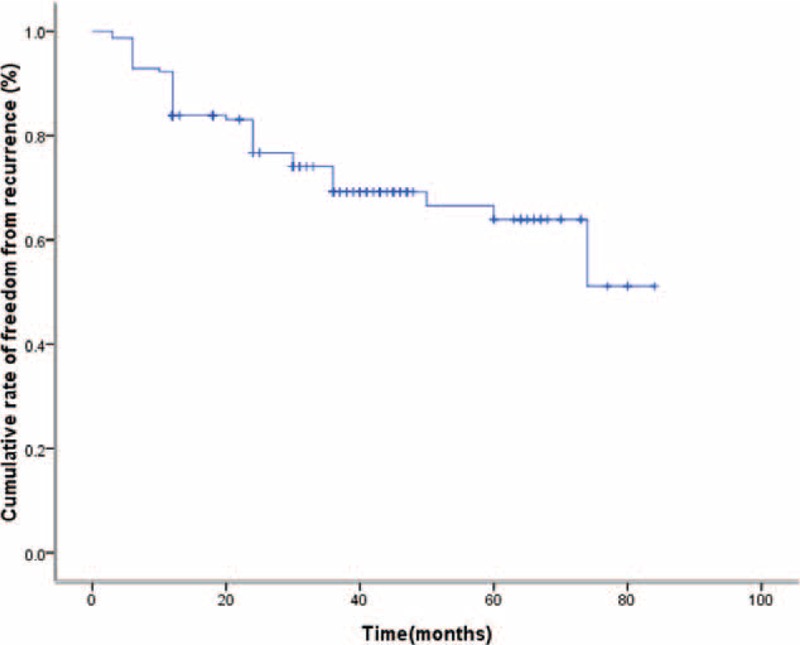
Cumulative rate of freedom from recurrence after USgHIFU for symptomatic adenomyosis. USgHIFU = ultrasound-guided high-intensity focused ultrasound.

Dysmenorrhea recurred in 45 (26%) patients. The age of these patients were 38.7 ± 5.3 years (range, 24–49 years). The median recurrence time was 12 months (range, 3–74 months). Of the 45 patients, 42 (93.3%) experienced dysmenorrhea recurrence within 3 years, whereas only 3 patients recurred after 3 years. The cumulative rate of freedom from recurrence according to Kaplan–Meier analysis was 72.9% after 36 months (Figure 2). Results of univariate analysis are shown in Table 3. A multivariate analysis was performed and 2 independent predictive factors were identified: the BMI (OR = 1.222, 95% CI = 1.079–1.381, *P* = 0.001) and the mean acoustic intensity (OR = 0.992, 95% CI = 0.986–0.998, *P* = 0.007). As for symptom recurrence, BMI is a risk factor, whereas the mean acoustic intensity showed a protective effect.

**TABLE 3:**
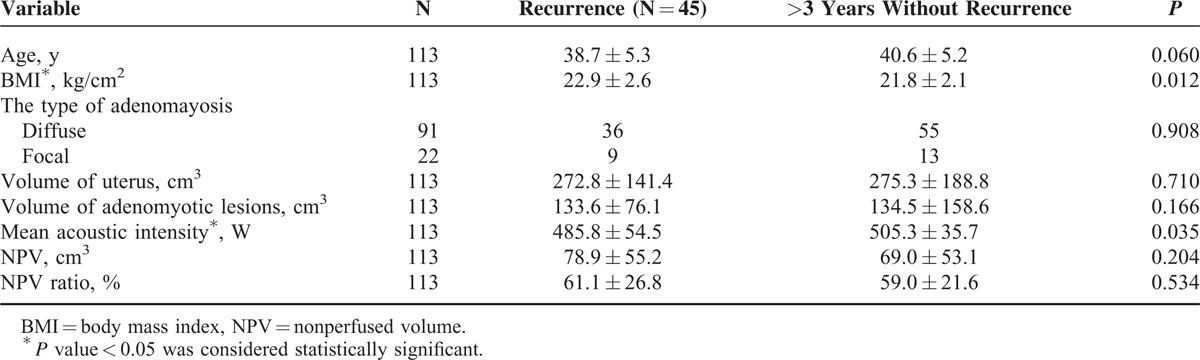
Univariate Analysis of Parameters Associated With Recurrence

**FIGURE 3:**
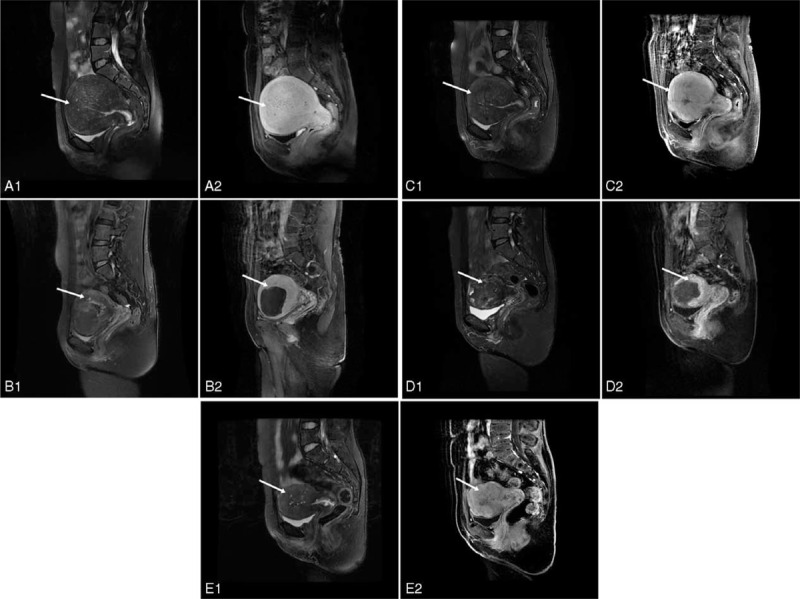
A 44-year-old woman who underwent twice USgHIFU ablation for symptomatic adenomyosis. (A1-A2) Sagittal view of MR image obtained before the first USgHIFU ablation showed diffuse junctional zone thickening of the uterus (arrows). The volume of the uterus was 581 cm^3^. (B1–B2) Sagittal view of MR image obtained 3 months after first USgHIFU ablation showed decreased size of the uterus. The volume of the uterus was 371 cm^3^. Arrows indicate nonperfusion area (183 cm^3^) after USgHIFU. (C1–C2) Sagittal view of MR image obtained 48 months after first USgHIFU ablation (before second USgHIFU ablation) showed enhancement of the treated area (arrows). The volume of the uterus was 319 cm^3^. (D1–D2) Sagittal view of MR image obtained 12 months after second USgHIFU ablation showed decreased size of the uterus. The volume of the uterus was 150 cm^3^. Arrows indicate the nonperfusion area (54 cm^3^) after treatment. (E1–E2) Sagittal view of MR image obtained 24 months after second USgHIFU ablation showed enhancement of the treated area (arrows). The volume of the uterus was 122 cm^3.^ After twice USgHIFU, the patient was asymptomatic until she was menopausal. MR = magnetic resonance, USgHIFU = ultrasound-guided high-intensity focused ultrasound.

A total of 31 patients whose dysmenorrhea recurred received additional treatments: of whom 7 had partial or complete hysterectomy, 3 had the levonorgestrel-releasing intrauterine system, another 7 had GnRH agonists or painkillers and the rest 14 patients had retreatment with USgHIFU ablation (Figure 3). Among the 14 patients treated with USgHIFU ablation, 12 (85.7%) achieved clinical success.

### Adverse Effects

No serious adverse effects occurred. According to SIR classification, 82(39.4%) patients recorded adverse effects belonged to SIR Type A category and no clinical intervention was needed. A total of 74 patients felt a mild to moderate pain in the treated area, and then disappeared 1 to 3 days after the treatment. Vaginal discharge appeared in 6 patients and then disappeared after 3 months. Numbness of the lower limbs were observed in 2 patients and the symptom was gradually relieved within 3 months after treatment. Two (1%) patients had I° burns on the abdominal skin which belonged to SIR Type B. They were treated with local dressing and the burns healed after 14 days.

## DISCUSSION

As for now, there is still no satisfactory uterus-conserving treatment for adenomyosis. Oral hormones and GnRH agonists used to interfere with endocrine function in patients, nevertheless, the symptoms may relapse after withdrawal.^16^ The limitation of the uterus-sparing surgery lies in the fact that the boundaries of lesions and normal myometrium are difficult to distinguish.^17^ Also, fibrotic scars resulted from uterus surgery may make the treatment impossible to repeat. In UAE therapy, the ovarian function may be affected by the embolization of bilateral uterine artery. USgHIFU is a powerful tissue ablation technique, of which repeat treatment can be easily accepted due to its noninvasive and no radiation characteristics.^18^ During the treatment, an ultrasound probe is used to provide real-time imaging for the targeted lesions. By moving the focus, the lesions are ablated conformally, resulting in coagulation necrosis of the lesions and leading to alleviation of adenomyosis symptoms.

Dysmenorrhea is a major symptom of adenomyosis. Therefore, the extent of dysmenorrhea relief after treatment can be used as an important indicator of the effectiveness of the treatment. In the present study of 208 patients, the VAS score of dysmenorrhea had decreased significantly after 3 months, with an NPV ratio of 57.4 ± 24.4% and a clinical success of 83.2%. This finding is in line with the results of Fukunishi et al,^19^ which were a 12.7% uterus volume reduction and a significant reduction in SSS at 6-month follow-up. Larger changes were also reported in another study.^8^ However, little has been published on outcomes beyond 3 years. In our study, the median follow-up was 3.5 years, with a minimum of 1.5 years and a maximum of 7 years. Pelage et al^20^ and Kim et al^21^reported that a sustained symptoms relief of the UAE treatment after 2 years and 4.9 years were 55.0% and 57.4%, respectively. However, 71.0% of the patients in our study were asymptomatic during follow-up, of whom 19 patients had been menopausal.

As the severity of adenomyosis symptoms correlates roughly with lesions extent^22^ and Wang et al^6^ suggested that a better pain relief may be attributed to the larger volume of necrosis, we thus hypothesis that a lager NPV ratio may lead to higher effective rate. The present study confirmed the hypothesis and a significant association between NPV ratio and treatment success was demonstrated. Zhou et al^5^ had found that patients with NPV ratios > 50.0% have better treatment outcomes in terms of reduction of symptoms. A lower cutoff point of 39.2% was determined to be predictive of clinical success in our study. The NPV ratio was closely related with 4 parameters: the type of adenomyosis, treated adenomyotic lesions volume, NPV, and mean acoustic intensity. Diffused lesions other than focal lesions, smaller treated adenomyotic lesions volume, lager NPV or higher acoustic intensity can result in a lager NPV ratio. This finding is in line with the results of Wang et al.^6^ Therefore, we believe that an important goal of this treatment is to achieve as high NPV ratio as possible with safety in priority. In addition, age is another factor that contributes to the clinical success. The group with age ≥40 years achieved a higher chance of clinical success rate than the group with age <40 years. The reason may be attributed to the higher level of oestrogen presented in younger women, which can lead to a stronger invasion of the lesions. Also, a relative conservative treatment due to fertility preservation in young patients may lead to clinical inefficiency among some patients with lower NPV ratio. These findings may help us to establish criteria for patient selection and outcome prediction in future USgHIFU ablation treatments.

All uterine-sparing therapies carry the recurrence risk of adenomyosis symptoms. In our study, dysmenorrhea recurred in 26% patients after 3.5 years. As for UAE, the recurrence rate was 38% to 46% after 2 to 5 years,^21,23^ which is higher. Additional treatments are necessary in the case of symptom relapse. A total of 31 patients in our study received additional treatments, and 14 of them chose USgHIFU ablation treatments due to its easy acceptance, achieving a clinical success rate of 85.7% (12/14). Of the 12 success cases, 2 patients were asymptomatic until they were menopausal.

This study has several limitations. First, a small number of patients lost contacts during the long term follow-up. Additionally, as some of the margins between adenomyosis and normal myometrium were indistinct on MRI, this may lead to the inaccuracy of the volume measurement. Furthermore, this result is only a single center study which requires a poly center study to support the conclusion.

In conclusion, USgHIFU ablation is an effective uterus-conserving treatment for symptomatic adenomyosis with an acceptable long-term success rate. A higher chance of clinical success can be achieved in patients with larger NPV ratio and older age, whereas higher BMI and lower acoustic power may result in a higher chance of recurrence. These factors are helpful in selecting patients who are suitable for USgHIFU and in predicting the durability of symptom relief.
